# Analyses of the ratio of ganglion cell-inner plexiform layer thickness to vessel density according to age in healthy eyes

**DOI:** 10.1371/journal.pone.0292942

**Published:** 2023-10-18

**Authors:** Woo-Hyuk Lee, Yong-Jin Na, Hyung-Bin Lim, Jae-Yun Sung, Jung-Yeul Kim, Min-Woo Lee

**Affiliations:** 1 Department of Ophthalmology, Gyeongsang National University Changwon Hospital, Changwon, Republic of Korea; 2 Department of Ophthalmology, Konyang University College of Medicine, Daejeon, Republic of Korea; 3 1.0 Eye Clinic, Daejeon, Republic of Korea; 4 Department of Ophthalmology, Chungnam National University Sejong Hospital, Sejong, Republic of Korea; Irrua Specialist Teaching Hospital, NIGERIA

## Abstract

**Purpose:**

To identify how the inner retinal layer and microvasculature change with age by analyzing the relationships of ganglion cell-inner plexiform layer (GC-IPL) thickness, vessel density (VD), and the ratio of these measurements with age in healthy eyes.

**Methods:**

Participants were divided into five groups according to age. The GC-IPL thickness, VD, and GC-IPL/VD ratio were compared among the groups. Linear regression analyses were performed to identify relationships of GC-IPL/VD ratio with age.

**Results:**

The average GC-IPL thicknesses were 84.84 ± 5.28, 84.22 ± 5.30, 85.20 ± 6.29, 83.29 ± 7.06, and 82.26 ± 5.62 μm in the 20s, 30s, 40s, 50s, and 60s age groups, respectively. The VDs were 20.94 ± 1.50, 21.06 ± 1.50, 20.99 ± 1.03, 20.71 ± 0.93, and 19.74 ± 1.73 mm^-1^ in the 20s, 30s, 40s, 50s, and 60s age groups, respectively. The GC-IPL/VD ratio was 4.05, 4.00, 4.06, 4.02, and 4.17 in each group, respectively, and the ratio of the 60s age group was significantly higher than that of other groups. In linear regression analyses, the GC-IPL/VD ratio was significantly associated with age in the participants aged ≥ 50 years (B = 0.014, P = 0.013), whereas it was not in the participants aged < 50 years (B = 0.003, P = 0.434).

**Conclusions:**

GC-IPL thickness and macular VD showed a tendency to decrease beginning in the 50s age group and the GC-IPL/VD ratio was significantly increased in the 60s age group. Additionally, the GC-IPL/VD ratio was positively associated with age in subjects aged ≥ 50 years, which implies a more pronounced decline over time in VD rather than GC-IPL thickness.

## Introduction

Optical coherence tomography angiography (OCTA) is an imaging device used to observe the retinal microvasculature noninvasively, which can visualize the fine vasculature in multiple retinal layers [1, 2]. It also provides quantitative parameters of the retinal microvasculature including vessel density (VD), perfusion density (PD), and foveal avascular zone (FAZ) area; such parameters are useful for the diagnosis and management of many retinal diseases [3]. These OCTA parameters tend to decrease with increasing age [4, 5]. Jo et al. [5] reported a decrease in parafoveal VD of 0.116% per year in healthy subjects. Yu et al. [4] also reported that parafoveal VD decreased with increasing age by 0.4% per year. If subjects show a change beyond these physiological changes in these OCTA parameters, the disease is suspected. Therefore, it is crucial to evaluate the patterns of physiological changes according to age.

Ganglion cell-inner plexiform layer (GC-IPL) thickness can be measured using optical coherence tomography (OCT) and is a useful biomarker of retinal impairment in glaucoma, optic neuropathy, and various retinal diseases. Additionally, previous studies reported that the reduction of GC-IPL thickness is associated with increasing age in healthy eyes [6–9]. As such, both GC-IPL thickness and VD decrease with age and they are known to be significantly correlated with each other. Previous studies found a significant relationship between GC-IPL thickness and VD in patients with systemic disease including diabetes mellitus or hypertension and healthy individuals [9–11]. Therefore, retinal microvasculature and anatomical structure would be closely related. Retinal atrophy may cause impairment of microvasculature, and impaired retinal perfusion can lead to reduce retinal thickness. In a situation where they are influenced by each other and decrease with age, analyzing their ratios according to age can help compare the degree of decline of two parameters or infer a sequential relationship. Understanding the relationship between the two indicators in more detail can be useful in identifying normal aging effects or changes caused by disease.

In this study, we investigated how the inner retinal layer and microvasculature change with age by performing analyses of GC-IPL thickness, VD, and their ratio according to age.

## Methods

### Subjects

This retrospective, cross-sectional study was approved by the Institutional Review Board/Ethics Committee of Chungnam National University Hospital, Daejeon, Korea (No. 2022-02-008) and adhered to the tenets of the Declaration of Helsinki. We reviewed the charts of participants who visited our retinal clinic for a floater, cataract, unilateral epiretinal membrane, macular hole, or intraocular lens dislocation between March 2018 and December 2020; all participants had fellow eyes without any ophthalmic disease. The inclusion criteria were age 20 years or older, spherical equivalent between -6 and +3 diopters, no media opacities that affect fundus imaging, and normal clinical ocular examination findings with no evidence of retinal pathologies. Written informed consent was waived due to the retrospective nature of the study. We obtained subject information regarding best-corrected visual acuity (BCVA), spherical equivalent, intraocular pressure, axial length, and parameters of OCT and OCTA. We divided the subjects into five groups according to their age (20s, 30s, 40s, 50s, and 60s). The exclusion criteria were history of systemic disease including hypertension and diabetes, history of retinal and neuro-ophthalmic disease and glaucoma, history of ocular trauma, presence of any ophthalmic disease, age > 70 years, BCVA < 20/25, axial length > 26.0 mm, and history of intraocular pressure > 21 mmHg. One eye was randomly selected for inclusion in the study if both eyes met the inclusion criteria.

### OCT and OCTA measurements

SD-OCT (Cirrus HD OCT 5000, version 10.0; Carl Zeiss Meditec, Dublin, CA, USA) was performed using 512 by 128 macular cube scanning protocols to measure GC-IPL thickness. The ganglion cell analysis algorithm automatically measured GC-IPL thickness by identifying the outer boundaries of the retinal nerve fiber layer and the inner plexiform layer of the macula using the three-dimensional information from the macular cube scan. The average and sectoral GC-IPL thicknesses were analyzed.

OCTA images were obtained with a Cirrus HD-OCT 5000 along with AngioPlex software (Carl Zeiss Meditec), using an 840 nm wavelength and 68,000/second A-scans. Sensitivity and accuracy are ensured by incorporating the optical microangiography (OMAG) algorithm and retinal tracking technology. We obtained 3 × 3 mm fovea-centered scan areas, and en face OCTA images were used to analyze all scans, which were automatically generated by the OMAG algorithm used in AngioPlex software. The 3 × 3 mm scan was identical to the Early Treatment of Diabetic Retinopathy Study (ETDRS) inner circles. The central area is a central circle of 1 mm diameter, the ring area is the average of four-quadrant sectors, and the full area is a 3mm inner circle of the ETDRS. The software automatically measured VD (the total length of perfused vasculature per unit area) of the superficial capillary plexus, which spanned from the internal limiting membrane to the IPL. Images were excluded if they exhibited fixation loss or incorrect foveal centration, segmentation errors, motion artifacts, or signal strength < 9.

### Statistical analysis

Demographic characteristics, OCT, and OCTA parameters were compared using one-way analysis of variance, followed by post-hoc Bonferroni tests. The chi-squared test was performed for the comparison of categorical data. Linear regression analyses were performed to evaluate the relationship between age and GC-IPL thickness and VD. They were also performed between age and GC-IPL/VD ratio for participants < 50 years, for participants ≥ 50 years, and for all participants, respectively. SPSS software (version 18.0; IBM Corp., Armonk, NY, USA) was used to perform all statistical analyses.

## Results

### Demographics

A total of 288 eyes of 288 participants were enrolled; 46 in their 20s, 36 in their 30s, 45 in their 40s, 75 in their 50s, and 86 in their 60s (Table 1). The mean age of all participants was 50 ± 15.0 years, and the number of male participants was 148 (51.4%).

**Table 1:** Demographics and clinical characteristics of the subjects.

	20s age group (n = 46)	30s age group (n = 36)	40s age group (n = 45)	50s age group (n = 75)	60s age group (n = 86)	P-value
Age (mean ± SD, years)	27.43 ± 1.57	35.74 ± 2.59	46.11 ± 2.83	56.17 ± 2.65	64.88 ± 2.94	< 0.001
Sex (male, %)	23 (50.0)	16 (44.4)	20 (44.4)	38 (50.7)	51 (59.3)	0.320*
BCVA (mean ± SD, logMAR)	-0.02 ± 0.03	-0.01 ± 0.07	-0.04 ± 0.07	-0.03 ± 0.06	-0.01 ± 0.06	0.196
SE (mean ± SD, diopter)	-1.24 ± 2.26	-1.07 ± 2.07	-1.44 ± 1.57	-0.56 ± 1.68	-0.45 ± 1.54	0.068
IOP (mean ± SD, mmHg)	14.86 ± 3.31	14.88 ± 2.80	15.02 ± 2.60	15.40 ± 3.15	14.84 ± 2.95	0.609
Axial length (mean ± SD, mm)	24.38 ± 0.59	24.29 ± 0.74	24.24 ± 0.62	24.12 ± 0.62	24.14 ± 0.60	0.105
Cup/disc ratio (mean ± SD)	0.50 ± 0.18	0.50 ± 0.15	0.52 ± 0.16	0.53 ± 0.15	0.54 ± 0.14	0.102
CMT (mean ± SD, μm)	255.70 ± 20.49	257.17 ± 15.12	252.09 ± 15.79	257.35 ± 24.66	259.24 ± 27.84	0.698

*Using chi-squared test.

BCVA, best-corrected visual acuity; SE, spherical equivalent; IOP, intraocular pressure; CMT, central macular thickness.

Demographic and ocular characteristics among the groups were not significantly different except for age. The central macular thicknesses were 255.70 ± 20.49, 257.17 ± 15.12, 252.09 ± 15.79, 257.35 ± 24.66, and 259.24 ± 27.84 μm in the 20s, 30s, 40s, 50s, and 60s age groups, respectively, which did not also differ significantly (P = 0.698).

### GC-IPL thickness and OCTA parameters in each group

The average GC-IPL thicknesses were 84.84 ± 5.28, 84.22 ± 5.30, 85.20 ± 6.29, 83.29 ± 7.06, and 82.26 ± 5.62 μm in the 20s, 30s, 40s, 50s, and 60s age groups, respectively (Table 2).

**Table 2:** Ganglion cell-inner plexiform layer thickness in each group with age.

	20s age group	30s age group	40s age group	50s age group	60s age group	P-value
Average	84.84 ± 5.28	84.22 ± 5.30	85.20 ± 6.29	83.29 ± 7.06	82.26 ± 5.62	0.097
Superior	87.04 ± 5.15	84.86 ± 7.69	85.72 ± 8.75	84.13 ± 7.63	83.78 ± 6.14	0.471
Superotemporal	84.43 ± 5.12	84.00 ± 6.67	82.67 ± 7.58	80.69 ± 8.29	80.24 ± 7.49	0.199
Inferotemporal	85.25 ± 4.77	84.86 ± 6.20	82.44 ± 8.04	81.25 ± 7.11	80.96 ± 8.17	0.090
Inferior	83.96 ± 5.23	82.86 ± 7.75	81.78 ± 8.78	80.81 ± 7.32	79.45 ± 6.47	0.384
Inferonasal	85.89 ± 4.81	85.50 ± 6.22	84.61 ± 7.50	83.38 ± 9.29	82.44 ± 7.82	0.587
Superonasal	88.11 ± 4.82	87.64 ± 8.43	87.61 ± 6.31	85.47 ± 8.20	84.11 ± 7.04	0.481

The 50s and 60s age groups showed the tendency of thinner average GC-IPL than younger age groups, but it was not statistically significant (P = 0.097). The GC-IPL sectoral thicknesses showed a trend similar to the average GC-IPL thickness. The average GC-IPL thickness was 83.22 ± 5.71 μm in male participants and 82.77 ± 6.46 μm in female participants, which was not significantly different (P = 0.321).

The full areas of VD were 20.94 ± 1.50, 21.06 ± 1.50, 20.99 ± 1.03, 20.71 ± 0.93, and 19.74 ± 1.73 mm^-1^ in the 20s, 30s, 40s, 50s, and 60s age groups, respectively, which was significantly different (P < 0.001) (Table 3).

**Table 3:** Optical coherence tomography angiography measurements in each group with age.

	20s age group	30s age group	40s age group	50s age group	60s age group	*P-value (post-hoc)
Vessel density						
Central	11.18 ± 3.13	10.63 ± 2.74	11.12 ± 2.23	10.93 ± 3.34	10.09 ± 2.91	0.179
Ring	22.12 ± 1.51	21.98 ± 2.27	22.13 ± 1.02	21.99 ± 0.76	21.05 ± 1.90	0.001 (20s, 30s, 40s, 50s > 60s)
Full	20.94 ± 1.50	21.06 ± 1.50	20.99 ± 1.03	20.71 ± 0.93	19.74 ± 1.73	< 0.001 (20s, 30s, 40s, 50s > 60s)

*P-value with Bonferroni post-hoc test.

In post hoc analysis, the 60s age group showed significantly lower VD than the other groups. The full area of VD was 20.76 ± 1.51 mm^-1^ in male participants and 20.64 ± 1.46 in female participants, which was not significantly different (P = 0.476).

### GC-IPL/VD ratio in each group and linear regression analyses to evaluate the relationship between age and GC-IPL, VD, and GC-IPL/VD ratio

**Fig 1:**
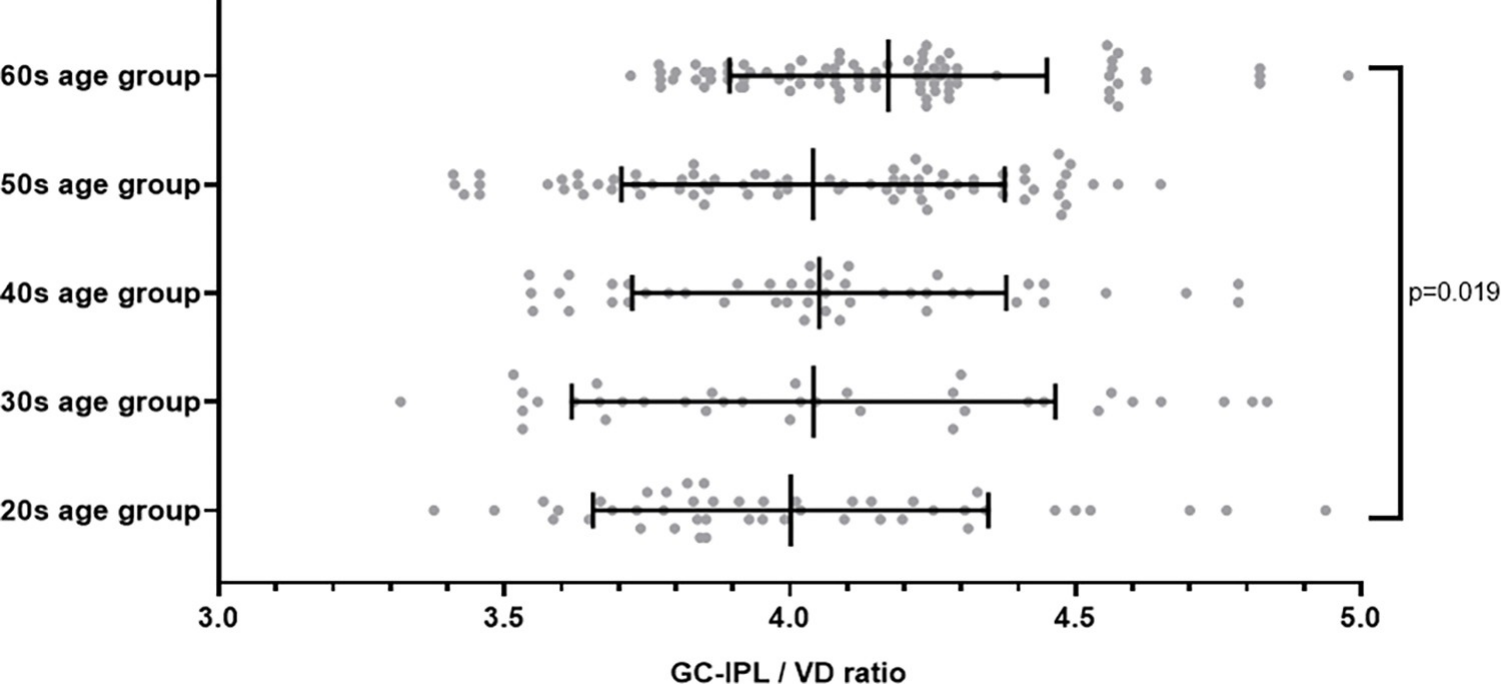
Scatter plots of the GC-IPL/VD ratio according to age.

The GC-IPL/VD ratios were 4.05, 4.00, 4.06, 4.02, and 4.17 in the 20s, 30s, 40s, 50s, and 60s age groups, respectively, which was significantly different (P = 0.019) (Fig 1). The average ratio remained relatively constant from the 20s to the 50s age group, but it increased significantly in the 60s age group (Bonferroni post hoc analyses: vs. 20s age group, P = 0.010; vs. 30s age group, P = 0.004; vs. 40s age group, P = 0.032; vs. 50s age group, P = 0.008).

**Fig 2:**
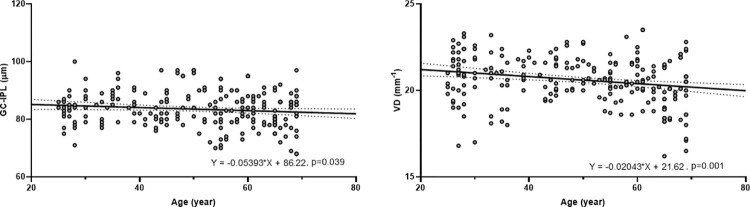
Scatter plots showing the relationships of GC-IPL thickness and VD with age.

**Fig 3:**
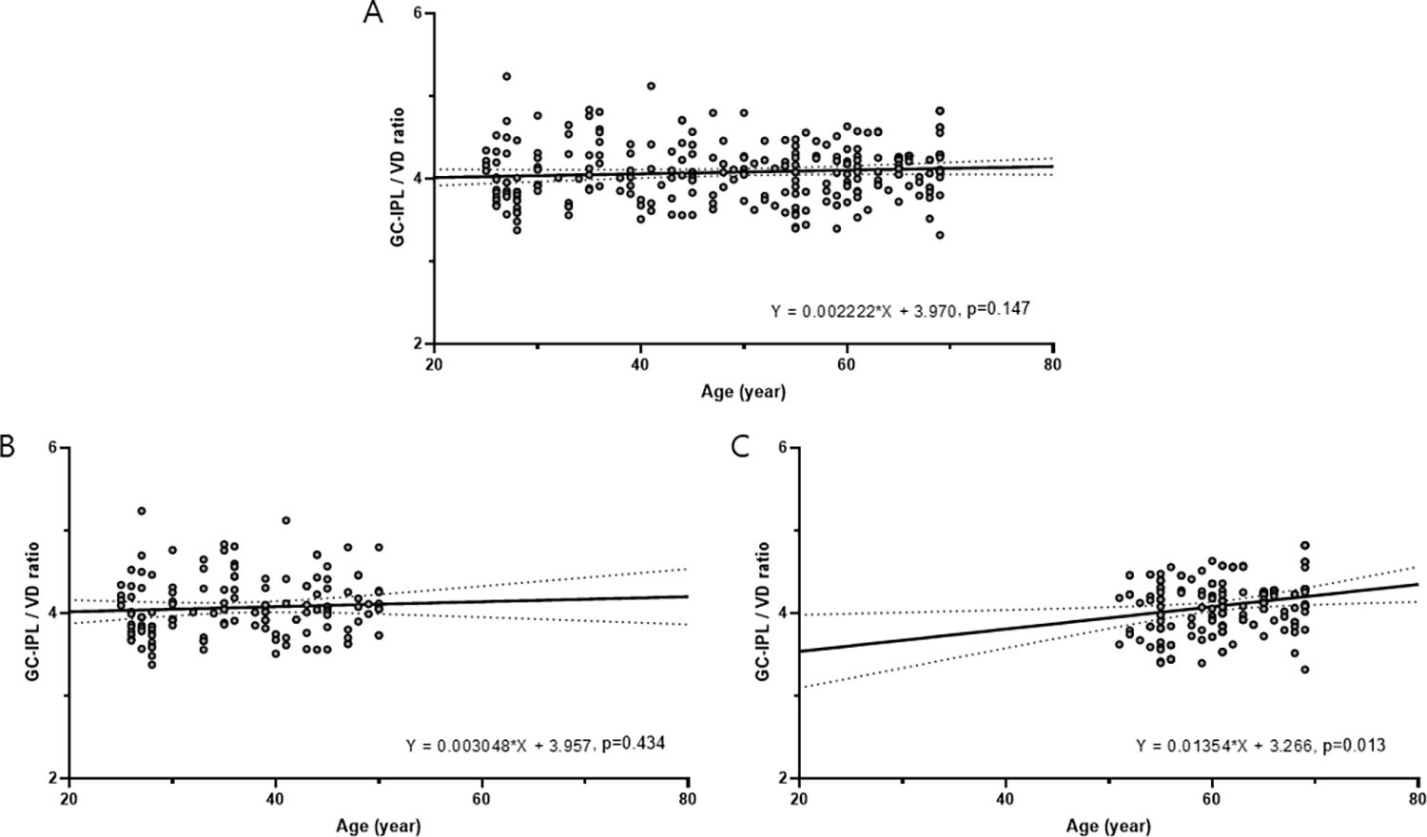
Scatter plots showing the relationships of GC-IPL/VD ratio with age. The GCIPL/VD ratio showed a tendency to increase with age but was not statistically significant (A). In participants aged < 50 years, the GC-IPL/VD ratio and age were not significantly associated in (B). However, the GC-IPL/VD ratio was significantly associated with age in older participants aged ≥ 50 years (C).

In linear regression analyses, GC-IPL thickness (B = -0.053, P = 0.039) and full area of VD (B = -0.020, P = 0.001) were significantly associated with age (Fig 2). The GC-IPL/VD ratio showed a tendency to increase with age but was not statistically significant (B = 0.002, P = 0.147). When participants were divided into younger and older groups based on a threshold age of 50 years (< 50 vs. ≥ 50 years), which was age beginning to show a decreasing trend in both GC-IPL thickness and VD, the GC-IPL/VD ratio and age were not significantly associated in the younger group (B = 0.003, P = 0.434) (Fig 3). However, the GC-IPL/VD ratio was significantly associated with age in the older group (B = 0.014, P = 0.013). Sex was not significantly associated with the GC-IPL thickness, (B = -1.553, P = 0.320), the full area of VD (B = -1.212, P = 0.476), and GC-IPL/VD ratio (B = -0.031, P = 0.545).

## Discussion

In this study, we investigated the GC-IPL thickness, macular VD, and GC-IPL/VD ratio in healthy eyes according to age and found that GC-IPL thickness and macular VD showed a tendency to decrease in the elderly. The GC-IPL/VD ratio remained constant among participants in their 50s or younger, but was significantly increased among participants in their 60s. In linear regression analyses, GC-IPL thickness and VD showed a negatively significant association with age, and the GC-IPL/VD ratio showed a positive association with age but was not statistically significant. However, the ratio was significantly associated with age in participants aged ≥ 50 years.

Lee et al. [6] reported that eyes with high myopia showed a -0.81 μm/year reduction rate of GC-IPL thickness in their 50s age. Lim et al. [7] reported that the reduction rate of GC-IPL in eyes with diabetic retinopathy was -0.987 μm/year, which was significantly higher than normal eyes. Another study found that the GC-IPL parameters were more valuable than the retinal nerve fiber layer parameters for glaucoma detection in eyes with parafoveal visual field loss [12]. As such, the GC-IPL thickness decreases in various ophthalmic diseases and could be a useful biomarker that plays a decisive role in the diagnosis and treatment of some ophthalmic diseases. In order to accurately analyze such changes in GC-IPL thickness, it is necessary to determine physiological changes in GC-IPL thickness according to age. Our study showed a significant association of age with GC-IPL thickness, and the thickness tended to be thinner beginning at the age of 50 years, although it was not statistically significant. Another study showed a marked thinner ganglion cell complex in 60s age group compared with younger age groups [5]. Therefore, such physiological changes should be considered in elderly subjects when interpreting changes in GC-IPL thickness.

Previous studies reported a decrease in macular OCTA parameters with age [4, 5]. Our study showed a significant association between age and macular VD, consistent with previous findings. This physiological decrease in macular microvasculature would be related to decreased cerebral blood flow which decreases with age approximately 0.50% per year [13]. Additionally, macular VD showed a relatively sharp decrease in their 60s, which was statistically significant. Although the exact mechanism can not be identified in this study, maintenance of the macular microvasculature may not be constant in older adults.

The GC-IPL/VD ratio was constant in subjects < 60 years of age, but was significantly increased among those in their 60s. This may be related to the marked decrease of macular VD in the 60s age group, which was more pronounced than the reduction of GC-IPL thickness. Notably, the macular VD represents the retinal microvasculature from the internal limiting membrane to IPL of the macular area, which contains the GC-IPL; therefore, macular VD and GC-IPL thickness are inevitably related to each other. Anatomical problems such as retinal atrophy may cause the impairment of retinal microvasculature and the reduced retinal blood flow can lead to retinal thinning. In the context of decreases in both GC-IPL thickness and macular VD, an increased GC-IPL/VD ratio implies disruption of the balance of anatomical structure with retinal perfusion, which had been maintained, via relatively prominent changes in the microvasculature. This imbalance appears to be more pronounced in individuals aged ≥ 60s years.

Both GC-IPL thickness and macular VD were negatively associated with age, and the GC-IPL/VD ratio showed a positive relationship with age, but it was not significant statistically. However, the GC-IPL/VD ratio was significantly associated with age in participants aged ≥ 50 years. The increasing ratio with age was caused by a more pronounced decrease in macular VD than a reduction of GC-IPL thickness, which implies that physiological changes in the microvasculature are more pronounced than anatomical changes in retinal structure over time. Additionally, these findings suggest the precedence of age-related changes in retinal microvasculature before the inner retinal thinning. Thinning of the anatomical retinal structure would be caused by a preceding decline in physiological retinal perfusion with age. Further longitudinal studies are necessary to identify the exact relationship.

This study had several limitations. First, some selection bias could be introduced inevitably by the retrospective nature of the work. A prospective longitudinal study would be ideal in the future to confirm our findings. Second, we could not analyze the parameters of the deep capillary plexus because the AngioPlex software provides only the parameters of the superficial capillary plexus automatically. Third, subjects older than 70 ages were not enrolled because of low BCVA or low quality of OCTA images by media opacity. Fourth, the number of cases included in each group varied greatly, which may cause some bias. The strength of our study was that few studies have reported the relationship between retinal structure, microvasculature, and age including various age groups, which we investigated. Additionally, we included high signal strength OCTA images over 8, which facilitated accurate analyses.

## Conclusions

GC-IPL thickness and macular VD showed a tendency to decrease in the elderly, and the GC-IPL/VD ratio was increased among subjects in their 60s, although it remained constant in younger subjects. The balance between retinal perfusion and the anatomical structure of the inner retina, which exhibits a close relationship, begins to deteriorate at approximately 60 years of age. Additionally, the GC-IPL/VD ratio was positively associated with age in subjects aged ≥50 years, which implies a more pronounced decline in retinal perfusion over time compared with retinal structural changes. These findings also suggest the precedence of physiological reduction of retinal microvasculature, which then causes gradual thinning of the inner retina with age.

## Supporting information

S1 Data (SAV)
